# Necrotizing Pancreatitis Due to Very High Triglyceride Level: A Case Report

**DOI:** 10.7759/cureus.69761

**Published:** 2024-09-19

**Authors:** Md Mostafizur Rahman, Mimnu Tasnim, Mingxin Li, Hariharan Devadas, Md Y Mamoon

**Affiliations:** 1 Biological Sciences, St. John’s University, New York, USA; 2 Internal Medicine, NYC Health + Hospitals/Queens (Queens Hospital Center), New York, USA; 3 Family Medicine, Efficient Medical Care PC, New York, USA; 4 Psychiatry, Creedmoor Psychiatric Center, New York, USA; 5 Medicine, St. George’s University, St. George, GRD

**Keywords:** alcoholism, hypertriglyceridemia, insulin, necrotizing pancreatitis, plasmapheresis

## Abstract

In the United States, acute pancreatitis is one of the most common gastrointestinal conditions that results in hospital admission. Necrotizing pancreatitis is a form of acute pancreatitis that can lead to various local and systemic complications. It is also associated with a high risk of mortality and morbidity without prompt intervention. In this case report, we discuss the case of a 33-year-old female with a history of alcoholism hospitalized with necrotizing pancreatitis due to hypertriglyceridemia. Our goal was to promptly identify the case by evaluating the signs and symptoms and intervening to prevent the associated complications. Our other objective was to change the diet and lifestyle of the patient to prevent the recurrence of necrotizing pancreatitis and readmission for the same reason.

## Introduction

Pancreatic necrosis is the hallmark of acute necrotizing pancreatitis (NP) and is associated with increased morbidity and mortality [1]. The etiologies of acute pancreatitis (AP) and NP are the same [2]. Hypertriglyceridemia (HTG) is the third most common identifiable cause of pancreatitis, after gallstones and alcoholism [3,4]. Moreover, medications such as 6-mercaptopurine or azathioprine and procedures such as endoscopic retrograde cholangiopancreatography are other potential causes of AP [5]. The diagnosis of NP depends heavily on imaging, particularly computed tomography (CT) scan and magnetic resonance imaging [1]. Infection, intestinal and biliary obstruction, bleeding, pseudoaneurysms, and venous thrombosis are some frequently occurring complications of NP [1]. While the general mortality rate for AP is about 5%, the mortality rate for NP is 17%, emphasizing the significance of early diagnosis and treatment [6].

In this article, we present a case of acute NP in a 33-year-old female with a history of alcoholism and a serum triglyceride (TG) level of 8,850 mg/dL. Although HTG is a well-known cause of NP, very severe HTG (>2,000 mg/dL) has been reported in only a few cases that resulted in AP or NP [7-9], and, ultimately, treatments with intravenous (IV) fluid, insulin, and plasmapheresis are needed for improvement.

## Case presentation

A 33-year-old Hispanic female with a past medical history of bulimia nervosa and major depressive disorder (MDD) presented to the emergency department with a complaint of sudden-onset, severe diffuse abdominal pain, described as squeezing and rated 10/10 in intensity, persisting for one day. The pain was sharp and worse in the periumbilical and right upper quadrant (RUQ), radiated to the back, and was exacerbated by movement but not alleviated by anything. The patient also complained of nausea and vomiting, loss of appetite, an inability to keep food down, and a low-grade fever lasting for one day. She denied headache, tinnitus, vertigo, jaundice, diarrhea, chest pain, shortness of breath, urinary symptoms, confusion, vision loss, syncope, fall, or trauma. She reported a history of bulimia nervosa from ages 14 to 18 and MDD for as long as she could remember but never received any treatment for these conditions. She denied having any past surgical history. Family history was significant for type 2 diabetes mellitus in both of her parents, and her father died from chronic kidney disease. She denied having any allergies and reported no history of smoking or using illicit drugs. She had an unclear vaccination history.

Upon admission, the patient stated that she was a single mother who immigrated from a country in South America to the United States at the age of 16. Her body mass index was 39.6 kg/m², and she had been unable to lose weight for years. She admitted to eating and drinking mostly carbohydrate-heavy junk foods, sodas, and juices. She endorsed having two to three drinks of vodka on weekdays and 10 drinks on weekends for the past 10 years.

Upon evaluation, she was anxious and distressed. Her pulse was 85 beats/minute, her blood pressure was 128/84 mmHg, and her temperature was 98.2°F. On abdominal examination, the abdomen was soft but tender in the RUQ and epigastric regions, with no evidence of ascites. The liver and kidneys were not palpable, and bowel sounds were present on auscultation. A psychiatric review revealed a history of fluctuating depressed mood, insomnia, low self-esteem, poor concentration, disturbed sleep, and feelings of hopelessness over the years. These symptoms met the Diagnostic and Statistical Manual of Mental Disorders, Fifth Edition (DSM-5) criteria for MDD. Additionally, she had a recurrent history of binge eating followed by compensatory purging, with a self-image closely tied to her weight, meeting the DSM-5 criteria for bulimia nervosa. Other systemic examinations showed no abnormalities. The laboratory findings are shown in Table 1.

**Table 1:** Laboratory findings. HDL: high-density lipoprotein; LFT: liver function test; ALP: alkaline phosphatase; ALT: alanine aminotransferase; PT: prothrombin time; INR: international normalized ratio

Parameter	Result			Normal range
	First day of admission	Second day of admission	Fifth day of admission	
Blood count				
Hemoglobin	9.2 g/dL	-	2.86 g/dL	12.0–16.0 g/dL
Hematocrit	28.5%	-	29.9%	36%–46%
Leukocytes	11.4 × 10^3^/mm^3^	-	6.39 × 10^3^/mm^3^	(4.5–11.3) × 10^3^/mm^3^
Neutrophils	9.6 × 10^3^/mm^3^ (84.7%)	-	-	(2.5–7.0) × 10^3^/mm^3^ (40–60%)
Serum lipase	1,145 U/L	-	224 U/L	0–160 U/L
Serum amylase	142 U/L	-	-	40–140 U/L
Serum lactate	2.5 mmol/L	-	-	<2 mmol/L
Triglyceride	8,850 mg/dL	-	552 mg/dL	<150 mg/dL
Cholesterol	903 mg/dL	-	142 mg/dL	<200 mg/dL
HDL	<3 mg/dL	-	13 mg/dL	40–60 mg/dL
Serum electrolytes				
Sodium (Na^+^)	-	118 mmol/L	142 mmol/L	136–146 mmol/L
Potassium (K^+^)	-	3.2 mmol/L	3.4 mmol/L	3.5–5.0 mmol/L
Chloride (Cl^-^)	-	103 mmol/L	102 mmol/L	95–105 mmol/L
Bicarbonate (HCO_3_^-^)	-	16 mmol/L	32 mmol/L	22–28 mmol/L
Serum calcium	-	8.8 mg/dL	-	8.4–10.2 mg/dL
serum albumin		4.2 g/dL	4.5 g/dL	3.5–5.5 g/dL
Serum glucose	-	157 mg/dL	-	<140 mg/dL
Serum creatinine	-	0.45 mg/dL	0.45 mg/dL	0.51–0.95 mg/dL
LFT				
Serum bilirubin (total)	-	0.9 mg/dL	1.0 mg/dL	0.1–1.0 mg/dL
ALP	-	68 U/L	76 U/L	25–100 U/L
ALT	-	22 U/L	30 U/L	10–40 U/L
PT	-	12.1 seconds	-	11–15 seconds
INR	-	1.1	-	0.8–1.2

**Figure 1:**
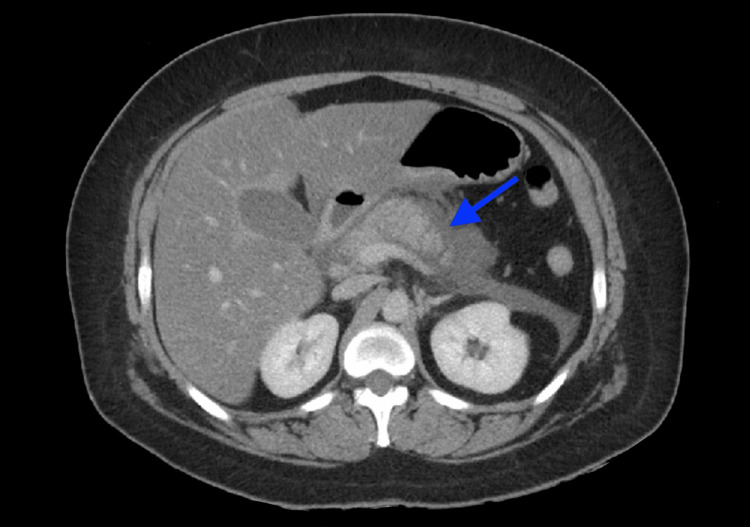
Computed tomography (CT) scan of the abdomen revealing areas of necrosis (blue arrow) indicative of acute necrotizing pancreatitis.

Further study with a chest X-ray was unremarkable. Moreover, a CT scan of the abdomen showed acute NP on the third day following the onset of symptoms (Figure 1). An ultrasonogram of the right upper quadrant showed mild sludge, moderate hepatomegaly, and steatosis.

**Figure 2:**
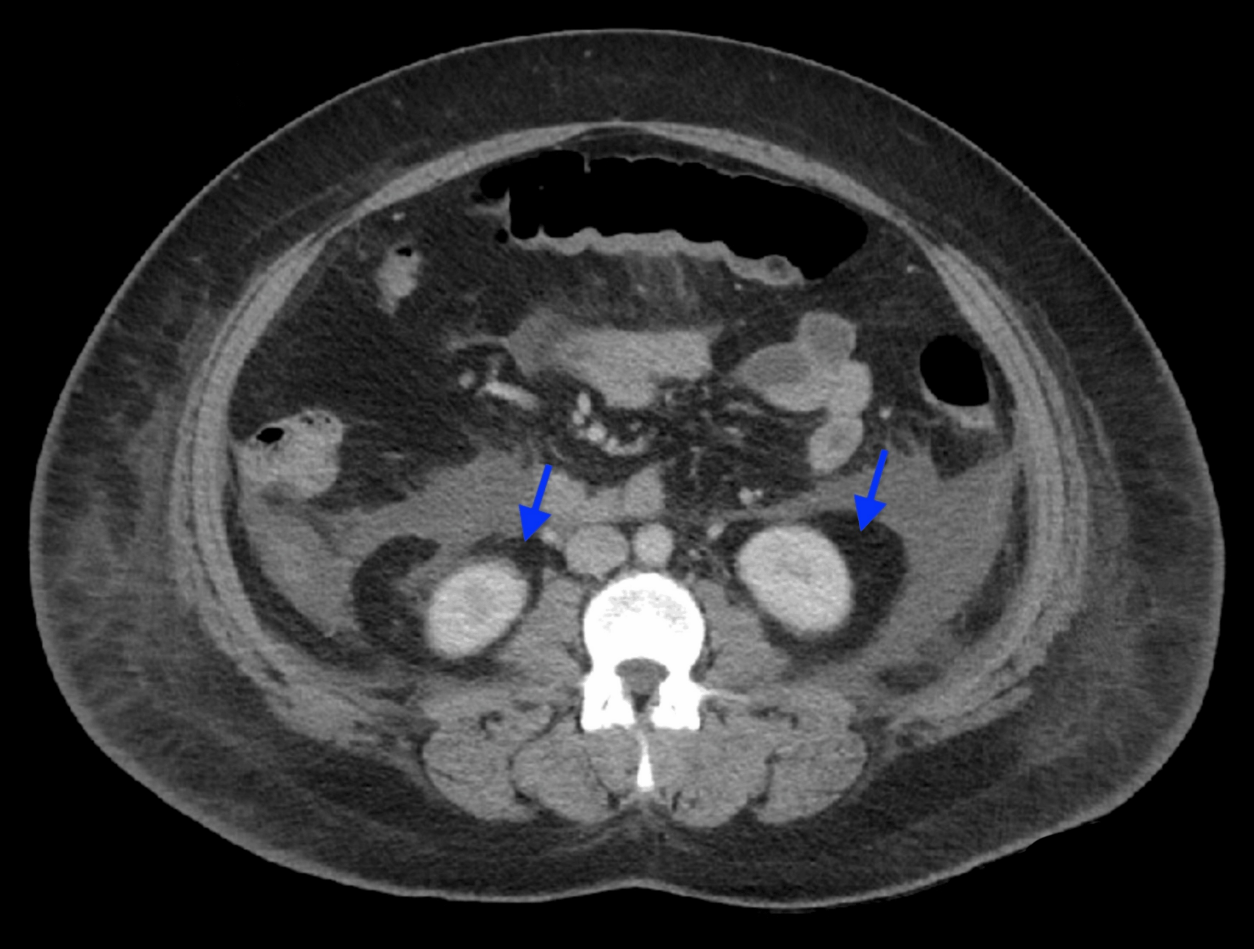
Computed tomography scan showing acute pancreatitis associated with extensive extra-pancreatic fluid in the right and left anterior pararenal spaces (blue arrows).

The patient was then admitted to the surgery intensive care unit for hemodynamic management and kept on nothing per oral. Aggressive IV fluid resuscitation, IV morphine, ondansetron, and empiric meropenem were started. The endocrinology team recommended starting IV insulin for HTG. On the second day, IV meropenem was stopped as there was no active sign of infection, and the white blood cell count decreased. On the third day of admission, the pain was improved. The patient underwent plasmapheresis as TG level (8,850 mg/dL) was >1,000 mg/dL and serum lipase level (1,145 U/L) was more than three times the upper limit of normal serum lipase (0-160 U/L). Then, she was transferred to the medical intensive care unit for further management. The patient was started on lactate ringer solution, and insulin drip and plasmapheresis were continued. Repeat TG decreased to 1,145 mg/dL. However, the patient developed acute hypoxic respiratory failure requiring a high-flow nasal cannula secondary to volume overload. Crackles were noted bilaterally, and IV furosemide was given. A follow-up CT of the abdomen on the sixth day revealed AP associated with small-volume ascites and extensive extra-pancreatic fluid (Figure 2). After the patient became hemodynamically stable, she was transferred to the step-down unit. A regular diet was started, and she tolerated it well. Foley’s catheter, Shiley’s catheter, and central line were removed. She continued to have some intermittent diffuse abdominal pain. IV morphine as needed was continued for pain management. Fenofibrate was started as per endocrinology’s recommendations. No surgical interventions were indicated, and the patient was hemodynamically stable and transferred to the medical floor for further management. All previous interventions except plasmapheresis were continued, and the patient only had mild RUQ pain. TG was improved to 552 mg/dL. Incentive spirometry was started. Subsequently, the patient was stable enough to be discharged home on oral medications with the advice of endocrinology, gastroenterology, and psychiatry follow-ups.

## Discussion

Alcoholism and HTG are the second and third most frequent causes of both AP and NP [4,10,11]. Although the precise mechanism of alcoholic AP remains unclear [10], patients who develop pancreatitis due to high TG levels are more likely to experience severe illness and have a greater chance of suffering from persistent organ failure [4]. Unfortunately, HTG is inadequately addressed commonly and undertreated as an AP risk factor, contributing to 1-9% of AP cases [11,12]. An elevated TG level of ≥500 mg/dL should prompt a high level of suspicion for AP, particularly when no other cause is evident. Furthermore, serum TG levels >1,000 mg/dL can trigger an episode of AP, although certain studies have indicated even higher thresholds. Clinical studies on the effect of HTG on AP severity have conflicting results. Few studies suggest mild TG elevations do not impact AP severity, but the role of TG levels >500 mg/dL remains unclear. Other studies indicate HTG patients may have more severe disease, with severity linked to TG levels [13,14].

In this case report, we review a case of NP in a patient with HTG (8,850 mg/dL) along with a history of alcoholism, class II obesity, and a family history of high lipid levels in her mother. The primary or familial HTG was initially thought to follow autosomal dominant inheritance due to monogenic mutations, but later studies suggested a polygenic origin [15]. Secondary HTG, which has a polygenic genetic basis, is exacerbated by environmental factors such as chronic alcohol abuse, obesity, diabetes, pregnancy, insulin resistance, chronic kidney disease, metabolic syndrome, hypothyroidism, and certain drugs [9,12,15]. In our case, a genetically predisposed individual developed high TG levels, exacerbated by environmental factors such as alcoholism, diet, and obesity. If left untreated, NP may have different complications, such as spontaneous hemorrhage, inflammation, abdominal compartment syndrome, and bowel ischemia [16]. In our case, we prevented any complications of NP by initiating early management.

This case also highlights the gut-brain connection, showcasing how untreated depression and eating disorders ultimately led to AP. The patient relied on alcohol to help her sleep and deal with stress. However, as a central nervous system depressant, alcohol aggravated her depression [17], for which she would resort to binging on carbohydrates. This not only caused uncontrolled weight gain but also elevated TG levels [18]. Gaining weight worsened her depression and sleep, which caused her to drink more, forming a vicious cycle. Moreover, she had never seen a physician since immigrating to the United States. Her reluctance could be explained by language and cultural barriers, a lack of economic resources, and fear of stigmatization due to obesity and psychiatric illness, especially in minority cultures. Had she seen a physician earlier, she would have had control over her alcoholism, bulimia nervosa, MDD, and HTG, which, in turn, would have prevented her from developing NP.

Various treatments have been proposed for managing pancreatitis due to HTG, including insulin infusions and plasmapheresis. Insulin enhances the synthesis of lipoprotein lipase, the key enzyme responsible for the hydrolysis of TG, thus reducing TG level, and apheresis methods such as plasmapheresis directly eliminate TG from blood [19]. A single plasmapheresis therapy can reduce TG levels by up to 70%, leading to significant clinical and laboratory improvements in patients with AP by minimizing further damage to the pancreas [9,20]. The timing of plasmapheresis is critical, with early intervention showing the greatest reduction in morbidity and mortality in several studies. However, this is expensive, and the morbidity and mortality benefit was not observed in a larger retrospective study, potentially due to delayed treatment [20]. In our case, the patient was treated with the Insulin drip immediately and then plasmapheresis on the third day. As a result, the TG level reduced dramatically from 8,850 mg/dL to 1,145 mg/dL and then eventually to 552 mg/dL. Maintaining TG levels at ≤500 mg/dL can prevent pancreatitis recurrence effectively if the cause is HTG. Treating HTG primarily involves diet, lifestyle adjustments, and controlling secondary factors, with lipid-lowering drugs as helpful long-term additions for managing TG levels [14]. In our case, the patient was counseled extensively on lifestyle modifications, such as abstaining from alcohol, following a low-carb diet, and exercising. A lipid-lowering agent, atorvastatin, was also prescribed.

## Conclusions

This case report demonstrates early diagnosis and treatment of NP, particularly when precipitated by severe HTG and with a history of alcoholism. Without early management, it can further deteriorate the patient’s condition and lead to complications. Blood tests and imaging are the most important ways to diagnose NP. IV fluid and insulin, followed by plasmapheresis, are the treatment of choice for necrotizing pancreatitis due to hypertriglyceridemia. Lifestyle modifications, especially abstaining from alcohol and following a low-carb diet, are key to preventing recurrences. A multidisciplinary approach, which involves prompt evaluation, tests, interventions, and patient education, is crucial for improving outcomes and reducing morbidity and mortality in patients with NP.
